# Ethnicity and thrombolysis in ischemic stroke: a hospital based study in Amsterdam

**DOI:** 10.1186/1471-2377-11-81

**Published:** 2011-06-30

**Authors:** Jonathan M Coutinho, Eva C Klaver, Yvo B Roos, Jan Stam, Paul J Nederkoorn

**Affiliations:** 1 Department of Neurology, Academic Medical Centre, Meibergdreef 9, 1105 AZ, Amsterdam, The Netherlands

## Abstract

**Background:**

Ethnic differences have been reported with regard to several medical therapies. The aim of this study was to investigate the relation between ethnicity and thrombolysis in stroke patients.

**Methods:**

Retrospective single-centre study. Patients admitted with an ischemic stroke between 2003 and 2008 were included. Ethnicity was determined by self-identification and stratified into white and non-white (all other ethnicities). The main outcome measure was the difference in thrombolysis rate between white and non-white patients. Logistic regression analysis was used to identify potential confounders of the relation between ethnicity and thrombolysis.

**Results:**

510 patients were included, 392 (77%) white and 118 (23%) non-white. Non-white patients were younger (median 69 vs. 60 years, p < 0.001), had a higher blood pressure at admission (median systolic 150 vs. 160 mmHg, p = 0.02) and a lower stroke severity (median NIHSS 5 vs. 4, p = 0.04). Non-white patients were significantly less often treated with thrombolysis compared to white patients (odds ratio 0.34, 95% CI 0.17-0.71), which was partly explained by a later arrival at the hospital. After adjustment for potential confounders (late arrival, age, blood pressure above upper limit for thrombolysis, and oral anticoagulation use), a trend towards a lower thrombolysis rate in non-whites remained (adjusted odds ratio 0.38, 95% CI 0.13 to 1.16).

**Conclusions:**

Non-white stroke patients less often received thrombolysis than white patients, partly as a result of a delay in presentation. In this single centre study, potential bias due to hospital differences or insurance status could be ruled out as a cause. The magnitude of the difference is worrisome and requires further investigation. Modifiable causes, such as patient delay, awareness of stroke symptoms, language barriers and treatment of cardiovascular risk factors, should be addressed specifically in these ethnic groups in future stroke campaigns.

## Background

Despite its proven efficacy, only a minority of patients with an acute ischemic stroke are treated with intravenous thrombolysis [1-3]. A delay in hospital presentation is the most important reason why this treatment is withheld from stroke patients [4,5]. Other factors associated with not receiving thrombolysis are female gender, older age, improving or too mild neurological deficit and admittance to hospitals that are non-academic, small, or located in areas with a low population density [6-9].

Various studies have shown consistently that the level of medical care is lower in ethnic minorities. Examples are reperfusion therapy for myocardial infarction [10], treatment of hypertension [11], and carotid endarterectomy [12]. Two studies have investigated the relation between ethnicity and thrombolysis for acute ischemic stroke in North-America [13,14]. Both found that black patients were significantly less likely to undergo thrombolysis than white patients. The magnitude of the difference varied considerably between the studies. Furthermore, because both were multi-centre studies, hospital related factors, in addition to ethnicity, may have attributed to the difference in thrombolysis rate.

The aim of the present study was to investigate the relation between ethnicity and thrombolysis in a single academic hospital with a multi-ethnic caption area.

## Methods

### Study population

We performed a hospital-based study in a large academic hospital in Amsterdam, the Netherlands. Patients admitted with an acute ischemic stroke between January 2003 and February 2008 were collected in the Academic Medical Centre (AMC) stroke registry. For the purpose of this study, additional data on ethnicity and stroke treatment were retrospectively collected from the hospital records and patients interviews. Amsterdam has a multi-ethnic population; 35% of all inhabitants have a non-Western ethnicity http://www.os.amsterdam.nl. In the South-Eastern part of the city, where the hospital is located, Black and Asian are the most common ethnicities.

The diagnosis of ischemic stroke was established by a neurologist or resident in neurology. Stroke severity was assessed using the National Institutes of Health Stroke Scale (NIHSS) [15]. Strokes were classified into subtypes according to the 'Trial of Org 10172 in Acute Stroke Treatment' (TOAST) criteria [16]. Symptom-to-door times and door-to-needle times (if applicable) were calculated from the medical records. Patients who arrived within 2.5 hours from symptom onset were deemed potentially eligible for thrombolysis. We reviewed medical records to obtain demographic data and baseline characteristics, and contacted patients by telephone to determine ethnicity and to complete missing data. Patients were called at maximally three different occasions. If these attempts failed, or when a patient had died, a questionnaire was sent to the general practitioner. Ethnicity was determined by self-identification and stratified into the following six groups: (1) white, (2) black, (3) Asian, (4) Turkish, (5) Moroccan and (6) other. For all further analyses, we classified the groups as white (1) and non-white (all other ethnicities). The study protocol was approved by the ethical review board of the Academic Medical Centre.

### Thrombolysis protocol

The thrombolysis protocol of the Academic Medical Centre is based on the study protocol of the National Institute of Neurological Disorders and Stroke (NINDS) trial and uses the same selection criteria [17]. Prior to thrombolysis, all patients undergo a CT-scan to rule out intracerebral haemorrhage. If thrombolysis within 3 hours of symptom onset is possible (changed to 4.5 hours in September 2008) and no contraindications are present, the patient receives intravenous alteplase in a dose of 0.9 mg per kilogram of body weight (maximum, 90 mg), 10 percent of which is given as a bolus followed by delivery of the remaining 90 percent as a constant infusion over a period of 60 minutes. The AMC thrombolysis protocol does not employ an age-limit, nor is thrombolysis withheld in patients with a severe stroke. When the blood pressure is above the upper limit (185 systolic or 110 diastolic), we generally monitor the blood pressure every 10 minutes. If it spontaneously drops below the limit, thrombolysis is performed. We never apply pharmacological reduction of the blood pressure prior to thrombolysis.

### Statistical analysis

Categorical data were analysed with a X^2 ^test and continuous data with a Mann-Whitney test. To identify potential confounders of the relation between ethnicity and thrombolysis, we used logistic regression analysis. First, univariate analysis was performed to determine the association between preselected variables and thrombolysis (model I). The following variables were selected: arrival at the hospital within 2.5 hours of symptom onset, age, gender, previous stroke and diabetes, blood pressure above 185 systolic or 110 diastolic, mild stroke (defined as an NIHSS < 5), severe stroke (defined as an NIHSS > 25) and oral anticoagulation use. Next, these variables were entered in a bivariate model together with ethnicity (model II). If the bivariable odds ratio (OR) differed by more than 5% from the univariate OR for the relation between thrombolysis and ethnicity, the variable was considered a confounder and included in multivariate analysis (model III).

## Results

From the AMC stroke database, 510 patients with an acute ischemic stroke were included; 392 (77%) white and 118 (23%) non-white. 364 out of the 510 patients (71%) were contacted by telephone to assess self-reported ethnicity and, sporadically, other missing values. Among non-white patients, black (53%) and Asian (37%) were the most common ethnic groups. Non-white patients were significantly younger than whites (69 vs. 60 years, table 1), had a higher body-mass index (25 vs. 27), a lower stroke severity (NIHSS 5 vs. 4) and a higher blood pressure at admission, both systolic (150 vs. 160 mmHg) and diastolic (82 vs. 90 mmHg). Small-vessel disease was the predominant stroke subtype in non-white patients, while large-vessel atherosclerosis and cardioembolic stroke were more common in white patients. Non-whites more frequently had diabetes (18 vs. 39%), whereas whites were more often previous or current smokers (64 vs. 47%). White patients more frequently used aspirin (19 vs. 10%) and oral anticoagulation (14 vs. 6%) at baseline.

**Table 1:** Baseline characteristics

	White (n = 392)	Non-white (n = 118)	P-value
**Ethnicity**			
Black	-	53%	
Asian	-	37%	
Turkish	-	3%	
Moroccan	-	1%	
Other	-	6%	
**Patient characteristics**			
Gender (% female)	45%	54%	0.08
Age	69 (59-79)	60 (49-72)	< 0.001
BMI	25 (23-27)	27 (24-31)	0.03
Systolic blood pressure	150 (135-176)	160 (140-186)	0.02
Diastolic blood pressure	82 (75-95)	90 (80-100)	0.003
NIHSS	5 (2-12)	4 (2-6)	0.04
**TOAST classification**			
Small-vessel disease	36%	59%	< 0.001
Large-vessel disease	40%	25%	
Cardioembolic stroke	15%	8%	
Other known cause	4%	4%	
Undetermined cause	6%	5%	
**Baseline medication use**			
Platelet inhibitors			
Aspirin	19%	10%	0.02
Dipyridamole	5%	5%	0.99
Clopidogrel	2%	1%	0.32
Oral anticoagulation	14%	6%	0.02
Antihypertensive	49%	43%	0.27
Antihyperlipidemic	26%	18%	0.08
Antidiabetic	15%	29%	< 0.001
**Cardiovascular risk factors**			
Hypertension	55%	62%	0.21
Untreated hypertension	23%	32%	0.15
Diabetes	18%	39%	< 0.001
Hypercholesterolemia	29%	34%	0.37
Smoking (previous or current)	64%	47%	0.003
Atrial fibrillation	14%	9%	0.11
Previous stroke	23%	20%	0.55

Continuous variables are given as medians with interquartile ranges between brackets. Abbreviations: BMI = body mass index; NIHSS = National institute of health stroke scale

In total, 85 out of 510 patients (17%) were treated with thrombolysis. Non-white patients with an acute ischemic stroke significantly less often received thrombolysis compared to white patients (7 vs. 18%, OR 0.34, 95% CI 0.15-0.71, table 2). There was no significant difference in the onset-to-treatment and door-to-needle times. Thrombolysis rates were similar among Asian and black ethnicities (both 7%). To identify explanatory variables for the difference in thrombolysis rate between white and non-white patients, we analysed the frequency of potential contraindications for thrombolysis in both ethnic groups (table 3). Non-white patients arrived at the hospital outside the time window for thrombolysis more often than whites (59 vs. 79%, p < 0.001). Non-white patients also more often had a blood pressure above 185/110 (17 vs. 28%). On the other hand, whites more frequently used oral anticoagulation at admission (14 vs. 6%) and more often were older than 80 (21 vs. 6%). There was no significant difference in the proportion of patients with a mild or severe stroke. In the subgroup of patients without an unconditional contraindication for thrombolysis (arrival at hospital after 2.5 hours from symptom onset, blood pressure above 185/110 or oral anticoagulation use), non-whites were still less often treated with thrombolysis, although the difference was no longer significant (45 vs. 29%, p = 0.12).

**Table 2:** Thrombolysis rate in white and non-white patients

	White (n = 392)	Non-white (n = 118)	p value
Intravenous thrombolysis	18%	7%	0.003
Time interval (thrombolysed patients)			
Onset-to-treatment time (minutes)	105 (80-147)	120 (91-165)	0.51
Door-to-needle time (minutes)	31 (21-55)	37 (27-114)	0.35

Continuous variables are given as medians with interquartile ranges between brackets. The data from the onset-to-treatment and door-to-needle times are derived from patients who received thrombolysis only.

**Table 3:** Potential contraindications for receiving thrombolysis

	White (n = 392)	Non-white (n = 118)	p value
Arrival hospital after 2.5 hours of symptom onset	59%	79%	< 0.001
Blood pressure higher than 185/110	17%	28%	0.02
NIHSS < 5	48%	56%	0.14
NIHSS > 25	1%	0%	0.44
Age > 80 years	21%	6%	< 0.001
Previous stroke and diabetes	6%	7%	0.88
Oral anticoagulation use	14%	6%	0.02

Variables which may potentially explain why patients were not treated with intravenous thrombolysis. NIHSS = National institute of health stroke scale

We performed logistic regression analysis to determine whether ethnicity was independently associated with the chance of receiving thrombolysis. First, the association between thrombolysis and each of the preselected variables was determined separately (table 4, model I). Then, each variable was entered in a model together with ethnicity, in order to determine the effect on the OR (model II). The variable 'arrival within 2.5 hours' had the largest effect on the association between ethnicity and thrombolysis (OR change from 0.34 to 0.60). Other variables that changed the OR by 5% or more in the bivariate analyses were: age, blood pressure above 185/110 and oral anticoagulation use. These four factors were therefore considered explanatory and were used in the multivariate analysis (model III). After adjustment for these confounders, there was still a trend towards a lower thrombolysis rate among non-white patients, but the association was no longer significant (adjusted OR 0.38, 95% CI 0.13-1.16, p = 0.09).

**Table 4:** Logistic regression analysis

	OR (95% CI)	P-value
**I. Univariate model**		
Ethnicity	0.34 (0.17-0.71)	0.004
Arrival hospital after 2.5 hours of symptom onset	0.033 (0.005-0.21)	< 0.001
Age	0.99 (0.98-1.0)	0.29
Gender	1.8 (1.1-2.9)	0.02
Previous stroke and diabetes	0.48 (0.14-1.6)	0.24
Blood pressure higher than 185/110	0.68 (0.35-1.3)	0.25
NIHSS < 5	8.1 (4.2-16)	< 0.001
NIHSS > 25	4.9 (0.30-79)	0.26
Oral anticoagulation use	0.15 (0.035-0.61)	0.008
**II. Bivariate model (ethnicity is added to each model)**		
Arrival hospital after 2.5 hours of symptom onset	0.60 (0.24-1.50)*	0.28
Age	0.30 (0.14-0.63)*	0.001
Gender	0.36 (0.17-0.74)	0.006
Previous stroke and diabetes	0.34 (0.17-0.71)	0.004
Blood pressure higher than 185/110	0.33 (0.15-0.71)*	0.004
NIHSS < 5	0.36 (0.16-0.78)	0.01
NIHSS > 25	0.34 (0.16-0.73)	0.006
Oral anticoagulation use	0.31 (0.15-0.64)*	0.002
**III. Multivariate model**	0.38 (0.13-1.16)	0.09

The univariate model (I) shows the odds ratio for receiving thrombolysis for the variables separately. Next, the association with ethnicity is analysed in the bivariate model (II), using the same preselected variables. Finally, in the multivariate model (III) the variables which significantly changed the odds ratio (age; blood pressure higher than 185/110; oral anticoagulation use; arrival at the hospital after 2.5 hours of symptom onset) were entered in a model together with ethnicity.

OR = odds ratio; CI = confidence intervals; NIHSS = National institute of health stroke scale; * indicates a 5% or greater change in odds ratio

## Discussion

Non-white patients were two-and-a-half times less likely to receive thrombolytic treatment than white patients. An important explanatory variable for this difference was a later arrival at the hospital of non-white patients.

Two studies previously investigated the relation between ethnicity and thrombolysis for acute ischemic stroke; both found that black patients were significantly less likely than white patients to receive thrombolysis [13,14]. For both studies, data were obtained from many different hospitals throughout the USA. This may provide a bias with regard to the observed treatment difference, due to the inter-hospital differences that exist in the use of thrombolytic treatment [7]. If those hospitals with lower thrombolysis rates provide care to areas predominantly inhabited by people from ethnic minorities, the reported treatment difference could in reality be the result of differences in hospital related factors, rather than ethnic factors. Adjustment for hospital factors, such as bed size and number of stroke admittances, as both studies did, might only partially correct this bias. Furthermore, in the study by Schwamm et al, adjustment for stroke severity and uncontrollable hypertension, two factors that influence the decision to use thrombolysis, was not possible. Finally, the study by Johnston et al was performed in 1999, only a few years after the introduction of thrombolysis for acute ischemic stroke. At that time some hospitals may still have been in the start-up phase, leading to treatment variations among hospitals. In contrast, the present study was carried out more than 10 years after the introduction of thrombolytic therapy in a single academic centre in Amsterdam. Historically, this hospital serves many different ethnic groups who live in the same relatively small area. There is no reason to assume that in this particular region access to medical services or to (public) transportation systems would vary for white and non-white patients. Patients are treated similarly upon arrival at the hospital. According to Dutch law, hospitals and doctors are obliged to provide emergency medical care, including thrombolysis, to any patient regardless of insurance status. We therefore assume that it is unlikely that the differences in treatment we identified are caused by physician biases regarding ethnicity. On the other hand, insurance status may have had an effect on whether a patient decided to come to the hospital, and how fast.

Our study has several limitations. First, some of the data were collected retrospectively, which increases the risk of an unknown bias. Second, the relatively small study size precluded any subgroup analyses regarding specific ethnic minorities. Finally, to eliminate hospital related factors, we performed a single-centre study. Consequently, the results may not be representative of other hospitals.

An important explanatory variable for the difference in thrombolysis rate we observed was that non-white patients more frequently arrived at the hospital outside the time window for thrombolysis. Several previous studies have investigated hospital arrival times of ethnic minorities with an ischemic stroke. Similar to our study, two of these also found that non-whites arrived at the hospital significantly later than white patients [18,19]. Smith et al, however, did not find such a difference [20]. Ethnic minorities generally have a lower level of awareness regarding stroke symptoms, as has been found in several studies [21,22]. Several campaigns, with extensive TV coverage, have tried to raise stroke awareness in the Netherlands, and instruct people to immediately dial 112 (emergency medical services) at the first symptom of stroke. Unfortunately, these campaigns may not have sufficiently reached people from ethnic minorities, possibly due to cultural differences or language barriers.

After adjustment for the potential confounders, there was still a trend towards a lower rate of thrombolysis among non-white patients. Therefore, other, unidentified factors may have been of influence. One potential factor could be language barriers among non-white, often immigrant groups. Such barriers hinder communication between physicians and patients or their relatives, which could lead to the inability to ascertain the moment of symptom onset, or cause delays in taking the patient's medical history. Several studies have indeed found that the presence of language barriers is associated with a lower level of health care [23,24]. Insufficient treatment of cardiovascular risk factors, particularly hypertension, may also be a reason why non-whites less often received thrombolysis. Patients with inadequately treated hypertension are more likely to have a blood pressure higher than 185/110, a contraindication for thrombolysis. This hypothesis is corroborated by the fact that in our study population there was a trend towards more untreated hypertensive patients among non-whites than whites. It is also supported by the aforementioned finding that black patients are less likely than white patients to receive medical treatments, including some that are important in the management of cerebrovascular disease [10-12].

## Conclusions

Our results indicate that non-white patients with an acute ischemic stroke significantly less often receive thrombolytic treatment than white patients, confirming results from two North American studies. We identified that a delay in presentation was one of the most important explanatory factors. The additional value of our study is that hospital related factors and patient health insurance status could be ruled out as a cause in our setting. The magnitude of the difference is worrisome and requires further investigation. Modifiable causes, such as awareness of stroke symptoms, language barriers, patient delay and treatment of cardiovascular risk factors, should be addressed specifically in these ethnic groups in future stroke campaigns.

## Competing interests

The authors declare that they have no competing interests.

## Authors' contributions

JMC, YBR, JS and PJN designed the study. JMC and ECK acquired the data. JMC and PJN interpreted the data. JMC and ECK wrote the first draft of the paper. All authors provided useful comments on earlier versions of this manuscript. All authors have approved the final version of the manuscript.

## Pre-publication history

The pre-publication history for this paper can be accessed here:

http://www.biomedcentral.com/1471-2377/11/81/prepub
